# Salvianic acid A ameliorates ox-LDL-induced ferroptosis in vascular endothelial cells by regulating TFRC

**DOI:** 10.1097/MD.0000000000045542

**Published:** 2025-10-31

**Authors:** Shiwen Lu, Lifei Yu

**Affiliations:** a Department of Cardiology, The Second Affiliated Hospital of Guangxi Medical University, Nanning, Guangxi, China.

**Keywords:** atherosclerosis, ferroptosis, salvianic acid A

## Abstract

Atherosclerosis (AS) is an inflammatory disease which can results in the development of various cardiovascular diseases. Salvianic acid A (SAA) has certain curative effect on AS. This study focused on the effect of SAA on ferroptosis in human aortic endothelial cells (HAECs) in AS. HAECs were exposed to oxidized low-density lipoprotein (ox-LDL) to construct an in vitro AS model. CCK8 and PI stain was used to detect cell viability. Iron content, reactive oxygen species (ROS) as well as malondialdehyde was measured to detect the occurrence of ferroptosis. Protein expression were detected by western blotting. Real-time quantitative polymerase chain reaction (RT-qPCR) was applied to determine transferrin receptor (TFRC) levels. ox-LDL exposed resulted in decreased cell viability, increased mortality, increased iron content, ROS and malondialdehyde levels, increased RAS protein expression and decreased GPX4 protein expression of HAECs. However, the appropriate concentration of SAA could rescue this result. Besides, in ox-LDL-treated cells, t ferroptosis expression regulatory protein TFRC was decreased after SAA treatment. Overexpression of TFRC reversed the rescue effect of SAA on ox-LDL-treated cells. This study demonstrates that SAA ameliorates AS by affecting ferroptosis, thus providing new insights into the mechanism of AS development.

## 1. Introduction

Atherosclerosis (AS) is considered to be a chronic inflammatory disease. It is a pathological process in which lipids accumulate in the inner wall of the artery and form local plaques. It is mainly mediated by inflammation and is accompanied by oxidative stress, which often leads to a variety of cardiovascular diseases.^[1,2]^ The pathogenesis of AS is very complex, involving smooth muscle cell proliferation, vascular endothelial cell injury, lipid metabolism disorders, platelet adhesion and aggregation, and foam cell formation.^[3]^ In addition, the occurrence and development of AS are also affected by family genetics, unhealthy lifestyle and psychological factors.^[4]^ At present, statins are mainly used in clinical treatment of AS, but there are some side effects.^[5]^ Therefore, further research on AS is urgently needed.

Ferroptosis is a novel type of programmed cell death caused by lipid peroxides discovered by Dixon et al,^[6]^ which can be triggered by a variety of small molecules such as erastin and sulfadiazine. The occurrence of ferroptosis requires intracellular iron accumulation, accumulation of lipid peroxides and reactive oxygen species (ROS).^[7,8]^ Different from other cell death, ferroptosis is mainly characterized by mitochondrial shrinkage, mitochondrial outer membrane rupture, mitochondrial cristae decrease or disappear,^[9]^ accompanied by glutathione (GSH) depletion and glutathione peroxidase 4 (GPX4) activity decrease.^[10]^ Studies have shown that ferroptosis is related to the occurrence and development of nerve injury, cardiovascular diseases, and other diseases.^[11–13]^ It is reported that the serum iron content in patients with severe AS is significantly higher than that in patients with normal, mild, and moderate coronary artery diseases,^[14]^ and the characteristics of lipid peroxidation and iron deposition in plaque of patients with advanced AS,^[15]^ suggesting that ferroptosis plays an vital role in AS.

**Figure 1.**
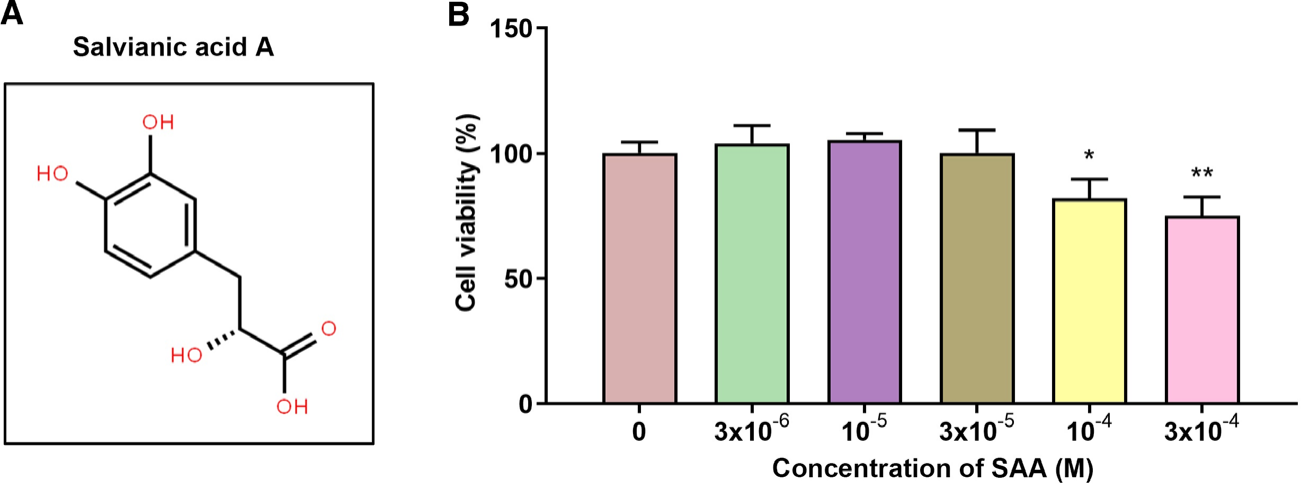
Selection of SAA concentration (A) Chemical structure of salvianic acid A. (B) Cell viability of HAECs treated with different concentration of SAA. All experiments were repeated 3 times (n = 3). *P < .05, **P < .01 versus control 0 M. HAEC = human aortic endothelial cells, SAA = salvianic acid A.

Chinese herbal medicine Salvia miltiorrhiza is the dry root and rhizomes of Salvia miltiorrhiza Bge, which is used in clinical treatment of cardiovascular diseases.^[16,17]^ Salvianic acid A (SAA) (Fig. 1A) is the main water-soluble component, chemically named β-(3, 4-dihydroxyl) phenyllactic acid, molecular formula C_9_H_10_O_5_, which is white long needle crystal. Studies have shown that SAA can reduce AS.^[18]^ For example, Yang et al^[19]^ found that SAA can inhibit or alleviate AS by inhibiting adhesion molecules and inflammatory cytokines in arterial endothelial tissue, and SAA can also resist oxidation and inhibit LPS-induced cell damage to protect vascular endothelial cells from AS.^[20]^ At present, there are few studies on SAA to alleviate and prevent AS.

In our study, we explore the clinical significance of SAA in preventing and treating AS by affecting ferroptosis of vascular endothelial cells, and to provide potential targets for AS treatment.

## 2. Materials and methods

### 2.1. Cell culture

Human aortic endothelial cells (HAECs) were purchased from the Type Culture Collection of the Chinese Academy of Sciences (Shanghai, P. R. China). Endothelial cell medium with endothelial cell growth factors, 5% fetal bovine serum, 100 U/mL penicillin, as well as 100 mg/mL streptomycin (Gibco, Massachusetts) was used to culture the cells at 37°C with 5% CO_2_. HAECs were treated with 25 µg/mL ox-LDL for 24 hours to establish an in vitro AS model. For SAA treatment, HAECs were cultured with different concentrations (3 × 10^−6^, 10^−5^, 3 × 10^−5^, 10^−4^, 3 × 10^−4^ M) of SAA for 24 hours, respectively.

### 2.2. Cell viability assay

HAECs, after resuspending, seeded in 96-well plates at 100 μl/well. Each well was added 10 μL CCK8 reagents (AmyJet Technology Co., Ltd.) and cultured for 4 hours in the 37°C environment. Microplate reader (Nanjing DeTie Experimental Equipment Co., Ltd.) was applied to detect absorbance values at 450 nm.

### 2.3. Cell death determination

HAECs was assessed using propidium iodide (PI) staining, as previously described, with minor modifications. Briefly, HAECs were seeded in 6-well plates at a density of 2 × 10⁵ cells per well and allowed to adhere overnight. The collected cell suspension was centrifuged at 1200 rpm for 5 minutes, and the cell pellet was resuspended in 500 μL of PI staining solution (50 μg/mL PI in phosphate buffer saline) supplemented with RNase A (100 μg/mL) to exclude DNA fragmentation effects. Samples were incubated in the dark for 30 minutes at room temperature. Fluorescence emitted from PI-stained nuclei was analyzed using a BD FACSCanto flow cytometer (BD Biosciences, San Jose). Results are presented as the percentage of PI-positive cells relative to the total cell population.

### 2.4. ROS, MDA, and iron levels determination

HAECs were homogenized with pre-cool phosphate buffer saline. The iron content was determined by iron assay kit (Bioassay Systems). ROS levels were detected with a fluorescence probe dichloro‐dihydro‐fluorescein diacetate (Jiancheng Biotech, Nanjing, China). MDA Assay Kit (Beyotime) was used to examine MDA content. All operations were performed strictly follow the instructions of the kits.

### 2.5. Western blot

Cell lysates were prepared using RIPA lysis buffer (Beyotime) supplemented with protease and phosphatase inhibitor cocktail. Following quantification of total protein concentration using the BCA Protein Assay Kit (Thermo Fisher Scientific), equal amounts of protein (30 μg per sample) were separated by SDS-PAGE on 10% to 12% polyacrylamide gels and subsequently transferred onto PVDF membranes (Millipore, Billerica). The membranes were blocked with 5% nonfat dry milk for 1 hour at room temperature and then incubated overnight at 4°C with primary antibodies specific to RAS (1:1000; Cell Signaling Technology), GPX4 (1:1000; Abcam), TFRC (1:1500; Abcam), or glyceraldehyde-3-phosphate dehydrogenase (1:5000; Abcam) as a loading control. After 3 washes with TBST, the membranes were incubated with horseradish peroxidase-conjugated secondary antibodies (1:5000; Cell Signaling Technology) for 1 hour at room temperature. Immunoreactive bands were visualized using an ECL detection system (Thermo Fisher Scientific), and images were captured using a ChemiDoc Imaging System (Bio-Rad, Hercules).

### 2.6. Real-time quantitative polymerase chain reaction (RT-qPCR)

Total RNA was extracted using the TRIzol reagent (Invitrogen; Thermo Fisher Scientific, Inc.). Reverse transcription and qPCR were performed using a BlazeTaq One-Step SYBR Green RT-qPCR Kit (with ROX) (QP071; GeneCopoeia, Inc., Maryland) on a SEDI Thermo Cycler controlled by the Control Bus Net software package (Wealtec Bioscience Co., Ltd., New Taipei City, Taiwan). All primers were designed and synthesized by Nanjing Genscript Biotech Co., Ltd., (Jianngsu, P. R. China). The results were analyzed using the 2^-ΔΔCt^ method. The sequences of the primers were as follows:

TFRC, F: 5′-AGTAGGAGCCCAGAGAGACGCTTGG-3′, R: 5′-CACTCAGTGGCACCAACAGCTCCAT-3′;

GAPDH, F: 5′-TCTTGTGCAGTGCCAGCCT-3′, R: 5′-TGAGGTCAATGAAGGGGTCG-3′.

### 2.7. Statistical analysis

All experiments were repeated 3 times. GraphPad Prism (version 7, GraphPad Software Inc.) was applied to analyze all data. And they were presented as mean ± SD. The Student *t* test was used to compare 2 groups’ differences, and the comparison among multiple groups used the analysis of variance followed by with Duncan post hoc test. *P* < .05 suggested a significant difference.

## 3. Results

### 3.1. Selection of SAA concentration

The chemical structure of SAA was showed in Figure 1A. In order to find an appropriate concentration of SAA, HAECs were treated with different dose of SAA, among which 3 × 10^-6^, 10^–5^, and 3 × 10^–5^ M of SAA had no effect on cell activity, while 10^–4^ and 3 × 10^–4^ M SAA significantly decreased the cell viability of SAA (Fig. 1B). This indicated that 10^–4^ and 3 × 10^-4^ M SAA had cytotoxicity, so we used SAA at 3 × 10^-6^, 10^–5^, and 3 × 10^–5^ M for subsequent experiments.

### 3.2. SAA treatment inhibited the ferroptosis of ox-LDL-treated HAECs

**Figure 2.**
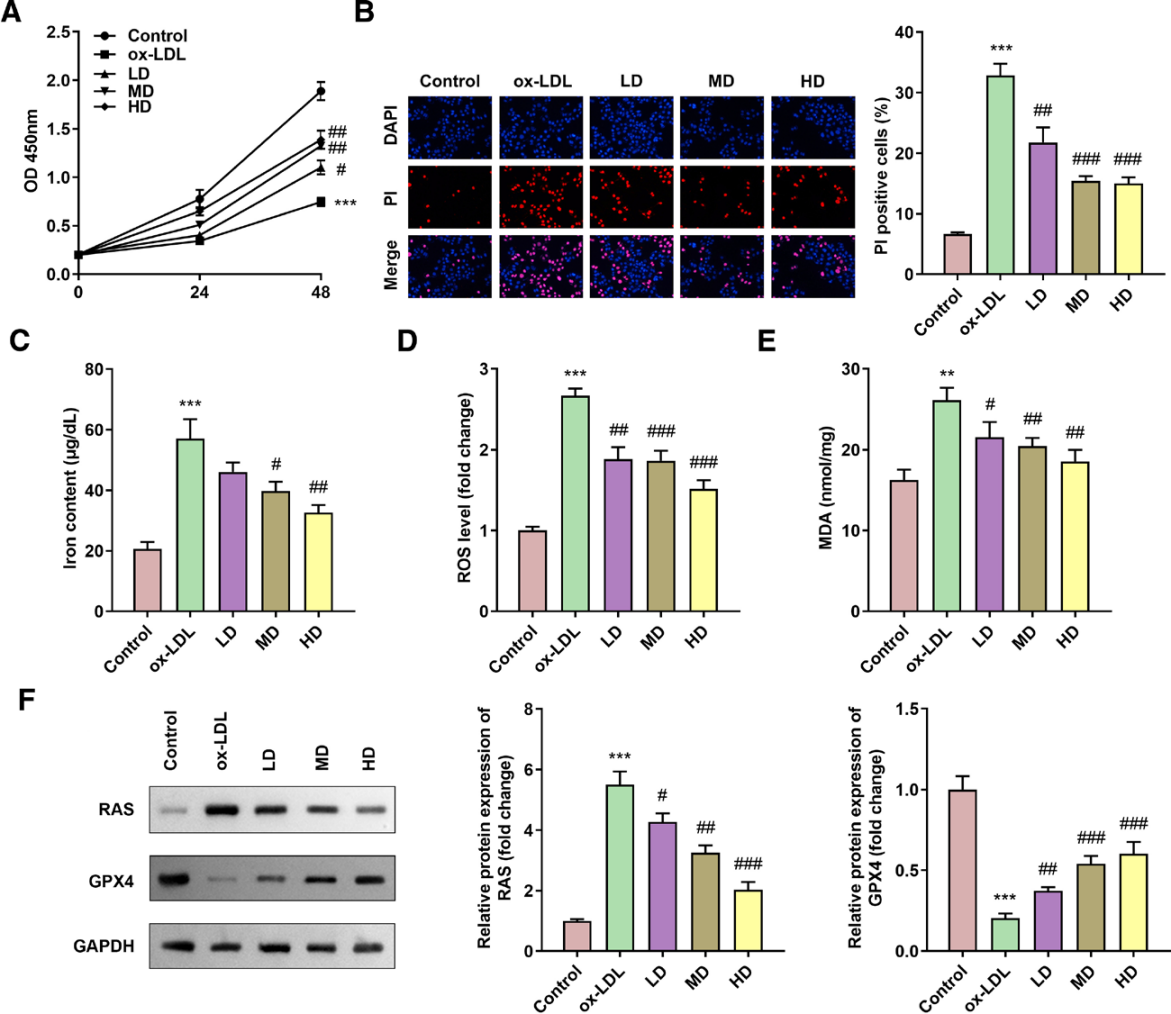
SAA treatment inhibited the ferroptosis of ox-LDL-treated HAECs (A) Cell viability of HAECs. (B) Cell death determined by PI staining in HAECs. (C) Iron content in HAECs. (D) ROS level in HAECs. (E) MDA in HAECs. (F) The protein expression of RAS as well as GPX4. All experiments were repeated 3 times (n = 3). *** *P* < .001 versus control. #*P* < .05, ##*P* < .01, ### *P* < .001 versus ox-LDL. HAEC = human aortic endothelial cells, ox-LDL = oxidized low-density lipoprotein, MDA = malondialdehyde, PI = propidium iodide, ROS = reactive oxygen species, SAA = salvianic acid A.

Compared with the cells in the ox-LDL treatment group, the cell viability increased (Fig. 2A) and the mortality decreased (Fig. 2B) after SAA treatment at different concentrations, and the effect of medium and high concentrations was better. In addition, iron content, ROS as well as MDA levels decreased after SAA treatment (Fig. 2C-2E), ferroptosis associated protein RAS expression decreased, and GPX4 expression increased (Fig. 2F). Similarly, medium and high concentration SAA had better effect, and high concentration (3 × 10^–5^) was used in the following experiments.

### 3.3. SAA treatment decreased the TFRC expression in ox-LDL-treated HAECs

**Figure 3.**
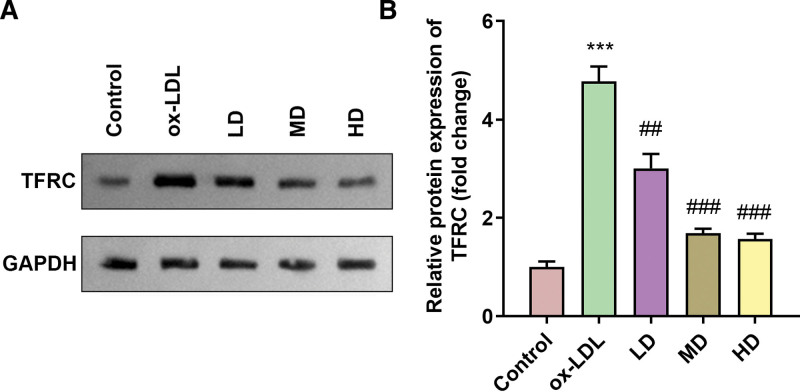
SAA treatment decreased the TFRC expression in ox-LDL-treated HAECs (A-B) The protein expression of TFRC. All experiments were repeated 3 times (n = 3). *** *P* < .001 versus control. ##*P* < .01, ### *P* < .001 versus ox-LDL. HAEC = human aortic endothelial cells, ox-LDL = oxidized low-density lipoprotein, TFRC = transferrin receptor.

Figure 3A-B showed that TFRC expression was significantly increased after ox-LDL treatment, whereas TFRC expression decreased after SAA treatment.

### 3.4. TFRC overexpression reversed the role of SAA in ox-LDL-treated HAECs

**Figure 4.**
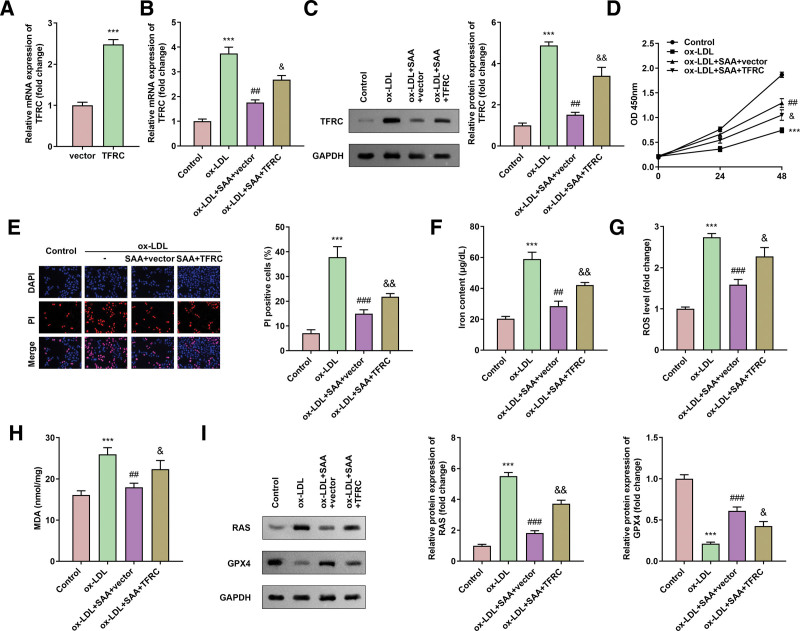
TFRC overexpression reversed the role of SAA in ox-LDL-treated HAECs (A and B) mRNA expression of TFRC. (C) The protein expression of TFRC. (D) Cell viability of HAECs. (E) Cell death determined by PI staining in HAECs. (F) Iron content in HAECs. (G) ROS level in HAECs. (H) MDA in HAECs. (I) The protein expression of RAS as well as GPX4. All experiments were repeated 3 times (n = 3). *** *P* < .001 versus control. ##*P* < .01, ### *P* < .001 versus ox-LDL. &*P* < .05, &&*P* < .01 versus ox-LDL + SAA + vector. HAEC = human aortic endothelial cells, MDA = malondialdehyde, ox-LDL = oxidized low-density lipoprotein, PI = propidium iodide, ROS = reactive oxygen species, TFRC = transferrin receptor.

We successfully overexpressed TFRC in cells by transfection (Fig. 4A). Compared with the cells treated with ox-LDL, the mRNA level and protein expression of TFRC in the cells exposed to SAA were significantly decreased, and TFRC could still be overexpressed in the cells treated with SAA (Figs. 4B and 4C). Compared with the ox-LDL-treated cells which rescued by SAA, overexpression of TFRC resulted in decreased cell viability and increased mortality (Fig. 4D and 4E). In addition, the overexpression of TFRC led to the increase of intracellular iron content, ROS and MDA levels (Fig. 4F-4H), and the increase of RAS protein expression and the decrease of GPX4 protein expression (Fig. 4I).

## 4. Discussion

AS is a disease that seriously endangers human health and is the pathological basis of many cardiovascular diseases.^[21]^ The traditional Chinese medicine Salvia miltiorrhiza has been reported to have significant effects in the prevention and treatment of cardiovascular diseases,^[22]^ and SAA is one of the active components of salvia miltiorrhiza. We revealed that SAA has a positive effect on AS in vitro, and safe concentration of SAA can rescue HAECs injury induced by ox-LDL. In addition, in ox-LDL-treated cells, SAA affected ferroptosis and reduced the expression of TFRC protein. Therefore, SAA may play a role in prevention and treatment of AS by reducing cell ferroptosis.

SAA is widely used in clinical practice of traditional Chinese medicine. For example, SAA protects the heart against ischemia reperfusion (I/R) injury by activating AKT/ERK1/Nrf2 signaling pathway.^[23,24]^ SAA reduces lipid deposition in Raw264.7 foam cells by balancing the expression of CD36 and ABCA1 proteins.^[25]^ In AS, SAA can significantly reduce lipid deposition in aorta and improve dyslipidemia to prevent the occurrence and development of AS.^[26]^ In this paper, SAA can enhance cell viability, reduce ROS and MDA levels, reduce iron content in cells, and increase RAS protein expression as well as reduce GPX4 protein expression, suggesting that SAA can prevent the occurrence and development of AS by inhibiting iron death in cells.

TFRC is a type II transmembrane glycoprotein that mediates intracellular iron uptake and regulates cell growth.^[27]^ Transferrin (TF) forms a complex by binding to the TFRC on the cell surface and transports iron into cells through cytocytosis, increasing the concentration of iron ions in cells, thereby enhancing the sensitivity of cells to ferroptosis induction.^[28,29]^ In this experiment, TFRC expression increased in ox-LDL-treated cells, but decreased after SAA was used. In addition, the rescue of SAA on cell damage was weakened by overexpression of TFRC in cells treated with ox-LDL as well as SAA.

In general, SAA can inhibit cell damage and ferroptosis caused by ox-LDL treatment, providing a potential target for the prevention and treatment of AS. However, there are still limitations in this study, as we have not conducted in vivo studies to comprehensively validate the therapeutic effect of SAA on AS. In addition, interplay between TFRC and other regulators (e.g., NCOA4, ACSL4, or HMOX1) is critical, these mechanistic details warrant dedicated follow-up studies to fully elucidate the regulatory hierarchy involving SAA, TFRC, and ferroptosis in AS.

## Author contributions

**Data curation:** Shiwen Lu.

**Formal analysis:** Shiwen Lu.

**Investigation:** Shiwen Lu.

**Resources:** Shiwen Lu.

**Supervision:** Lifei Yu.

**Validation:** Lifei Yu.

**Visualization:** Lifei Yu.

**Writing – original draft:** Shiwen Lu, Lifei Yu.

**Writing – review & editing:** Shiwen Lu, Lifei Yu.
